# Gut–Liver Axis and Non-Alcoholic Fatty Liver Disease: A Vicious Circle of Dysfunctions Orchestrated by the Gut Microbiome

**DOI:** 10.3390/biology11111622

**Published:** 2022-11-06

**Authors:** Salvatore Pezzino, Maria Sofia, Gloria Faletra, Chiara Mazzone, Giorgia Litrico, Gaetano La Greca, Saverio Latteri

**Affiliations:** Department of Surgical Sciences and Advanced Technologies “G. F. Ingrassia”, Cannizzaro Hospital, University of Catania, 95126 Catania, Italy

**Keywords:** gut microbiome, obesity, non-alcoholic fatty liver disease, dysbiosis, gut–liver axis

## Abstract

**Simple Summary:**

Non-alcoholic fatty liver disease (NAFLD) is a widespread disease associated with metabolic disorders, such as obesity. In recent years, there has been increasing evidence in favor of the relationship between the gut microbiome and NAFLD. In this review, we examine the impact of the altered gut microbiome on the pathophysiology of NAFLD. We focused on the pathological mechanisms and the role of gut–liver axis disruption—mediated by gut microbiome dysbiosis—on NAFLD development.

**Abstract:**

Non-alcoholic fatty liver disease (NAFLD) is a prevalent, multifactorial, and poorly understood liver disease with an increasing incidence worldwide. NAFLD is typically asymptomatic and coupled with other symptoms of metabolic syndrome. The prevalence of NAFLD is rising in tandem with the prevalence of obesity. In the Western hemisphere, NAFLD is one of the most prevalent causes of liver disease and liver transplantation. Recent research suggests that gut microbiome dysbiosis may play a significant role in the pathogenesis of NAFLD by dysregulating the gut–liver axis. The so-called “gut–liver axis” refers to the communication and feedback loop between the digestive system and the liver. Several pathological mechanisms characterized the alteration of the gut–liver axis, such as the impairment of the gut barrier and the increase of the intestinal permeability which result in endotoxemia and inflammation, and changes in bile acid profiles and metabolite levels produced by the gut microbiome. This review will explore the role of gut–liver axis disruption, mediated by gut microbiome dysbiosis, on NAFLD development.

## 1. Introduction

The term “gut microbiome” refers to the large number of commensal bacteria that live in the human digestive tract, the majority of which are anaerobic bacteria, and which collectively constitute between 500 and 1000 distinct species [1]. The predominant gut microbial phyla include *Firmicutes*, *Bacteroidetes*, *Actinobacteria*, *Proteobacteria*, *Fusobacteria*, and *Verrucomicrobia*, with *Firmicutes* and *Bacteroidetes* accounting for 90% of the gut microbiome [2,3]. These microbes are crucial for maintaining the integrity of the mucosal barrier function, as well as for the absorption of nutrients and the maintenance of energy balance [1,4]. Gut microbiome dysbiosis is linked to a variety of luminal and extraintestinal diseases, including metabolic diseases such as obesity [5] and chronic liver diseases [6,7]. Mucosal integrity, immunological function, and metabolism in the host can all be affected by dysbiotic microbiome [8,9]. The gut–liver axis refers to the communication and feedback loop between the digestive system and the liver. The liver is the first site in the body where bacteria and microbial components, as well as other endogenous and foreign toxins found in portal blood, are detected, and it provides the initial immunological and hormonal response to these molecules [10]. Furthermore, the interactions that take place between the intestine and the liver are mutually beneficial; the hormones, inflammatory mediators, and products of digestion and absorption all have a direct impact on the function of the liver [10,11,12]. The term “non-alcoholic fatty liver disease” (NAFLD) refers to a disorder that is produced by the deposition of fat within the liver cells even in the absence of alcohol usage [13]. It comprises a wide range of liver damage, from basic steatosis to non-alcoholic steatohepatitis (NASH), which is defined histologically by hepatocyte injury, inflammation, and varying degrees of fibrosis [7,14]. In most cases, these pathological conditions are associated with obesity or overweight [7,14], which are recognized risk factors for the onset of NAFLD [15]. Epidemiological studies showed that for people who are overweight, the incidence of NAFLD rises to between 22.5% and 44.0% [15], while in obese individuals, the prevalence of NAFLD can even reach 90% [16]. NAFLD develops into NASH in 20–30% of patients, with its consequences of liver scarring, cirrhosis, and liver cancer [15,17,18]. 

**Figure 1:**
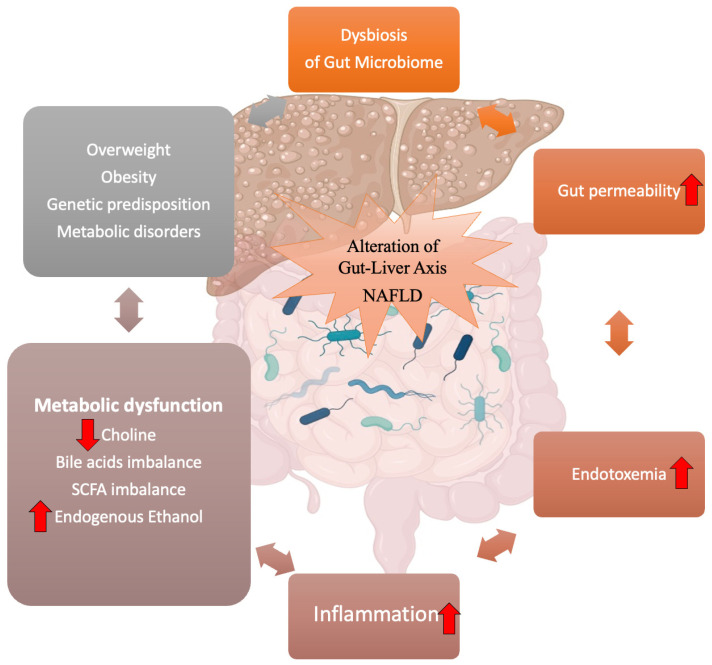
Gut–liver axis and NAFLD: a vicious circle of dysfunctions orchestrated by the gut microbiome. Alteration of the gut–liver axis is characterized by several pathological mechanisms, such as the impairment of the gut barrier and consequent increase of the intestinal permeability which result in endotoxemia and inflammation, and changes in bile acid profiles and metabolite levels (increasing of endogenous ethanol, reduction of choline levels, dysregulation of SCFA metabolism) produced by the gut microbiome. Gut microbiome dysbiosis has a prominent role in the disruption of the gut–liver axis. Created with https://BioRender.com (accessed on 10 October 2022) and modified with Microsoft PowerPoint v.16. SCFA: short-chain fatty acid; NAFLD: non-alcoholic fatty liver disease.

Although the exact cause of NAFLD is unknown, there is accumulating evidence from both preclinical and clinical studies suggesting that the gut–liver axis disruption plays a pivotal role in NAFLD pathogenesis and that gut dysbiosis is a determinant for the development of this dysfunction [19,20]. Alteration of the gut–liver axis is characterized by several pathological mechanisms, such as the impairment of the gut barrier and the increase of the intestinal permeability which result in endotoxemia and inflammation, and changes in bile acid profiles and metabolite levels produced by the gut microbiome (Figure 1). 

The purpose of this review is to provide an update of recent evidence on the gut–liver axis’ involvement in the modulation of NAFLD pathogenesis, with a focus on the impact of gut microbiota dysbiosis.

## 2. Gut Microbiome Dysbiosis in NAFLD and Obesity

Various studies have associated gut microbiome dysbiosis to the pathogenesis of NAFLD and obesity [5,6,21]. NAFLD has been linked to a different proportion between several gut microbe phyla, and research using animal models may provide some insight into this relationship. Research on NAFLD models showed conflicting findings about the bacterial species involved; a variety of changes in the relative abundances of *Bacteroidetes*, *Firmicutes*, and *Ruminococcus* have been observed [22,23,24,25,26,27,28]. These variations may reflect inherent differences between the various knock-out animals and deleted gene models studied. Nevertheless, the observation of hepatic steatosis development in germ-free mice after receiving an NAFLD-prone microbiome suggests that the propensity to develop NAFLD can be transmitted via gut microbiota fecal transplantation and provides direct proof of the relevance of gut microbiome dysbiosis for the onset of this pathology [29,30]. Moreover, induction of a healthy gut microbiome protects against the development of NAFLD in animals, as demonstrated in two studies where the generation of favorable changes to the gut microbiome with the probiotic *Bifidobacterium pseudocatenulatum* lowered the chance of developing NAFLD in mice [31,32]. 

**Figure 2:**
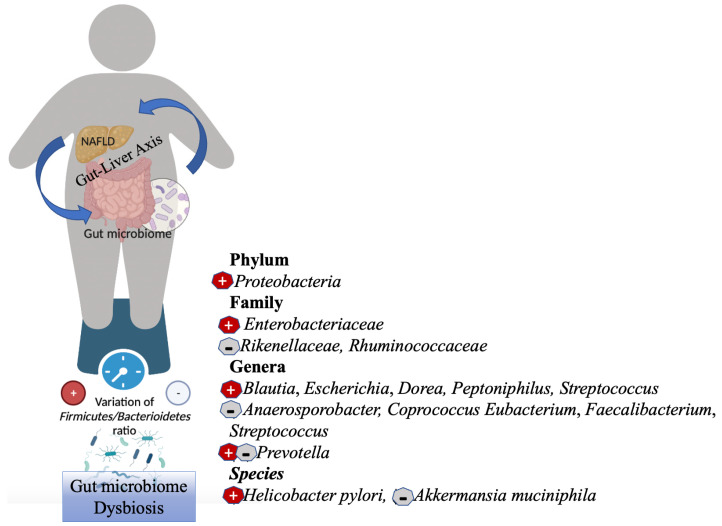
Gut microbiome dysbiosis in NAFLD. NAFLD is closely related to metabolic diseases such as obesity. Dysbiosis in NAFLD is characterized by a decrease in total bacterial diversity and richness, as well as in general by a shift toward a community composed of more *Firmicutes* and fewer *Bacteroidetes*. Meaning of the symbols in the figure: +, increased abundance; −, decreased abundance. Created with https://BioRender.com (accessed on 10 October 2022) and modified with Microsoft PowerPoint v.16.

Numerous clinical investigations have analyzed the gut microbiome of humans at different NAFLD stages. Overall, the gut microbiome of patients with NAFLD differs from that of healthy controls at the phylum level, with an increase in the number of *Proteobacteria* and *Firmicutes* and a decrease in the abundance of *Bacteroidetes* [33,34,35,36] (Figure 2).

As seen in scientific research using animals, in patients affected by NAFLD some studies found an increase in the number of *Bacteroidetes* and a reduction in the abundance of *Firmicutes* [37,38,39,40], while others found the opposite [41,42]. However, it must be considered that the *Firmicutes*/*Bacteroides* ratio is a relatively fair estimate due to the enormous diversity of microorganisms within each of these phyla. The composition of the gut microbiome may vary throughout demographic groups and stages of NAFLD, making it difficult to make definitive or causal statements. 

A recent comprehensive review and meta-analysis on compositional alterations of the gut microbiome in patients affected by NAFLD found a characteristic compositional pattern of fecal microbiome constituted by an increase in *Escherichia*, *Prevotella*, and *Streptococcus*, and a decrease in *Coprococcus*, *Faecalibacterium*, and *Ruminococcus* [43]. The meta-analysis disclosed also that the body mass index (BMI) may contribute to the abundance change of *Faecalibacterium* and *Prevotella* in patients with NAFLD compared to the healthy controls, and that the abundance changes of *Streptococcus* and *Faecalibacterium* are indicators of increased systemic inflammation and therefore a good index of progression of the pathology [43]. Recent species-specific studies have identified particular bacteria with possible implications for obesity and NAFLD development. *Akkermansia muciniphila*, a Gram-negative bacterium that degrades mucin, is one of them. *A*. *muciniphila* was found to correlate favorably with a better metabolic condition in mice and in human investigations with obese and diabetic participants [44]. *Helicobacter pylori* is usually linked to a variety of digestive diseases [45]. Recent studies have identified a significant frequency of NAFLD in *H. pylori*-positive patients, indicating a link between the presence of this bacteria and the development of fatty liver [46,47]. 

Obesity is closely related to NAFLD [16,48,49]. NAFLD development is influenced by dysbiosis caused by obesity [50]. Obesity remains a major public health problem in Western societies, where its prevalence has increased considerably in recent years [51,52]. The main causes that contribute to the spread of obesity are thought to be sedentary lifestyles and excessive food intake [53,54], which induce a state of nutritional imbalance, especially in genetically predisposed individuals [53,54]. As for NAFLD, obesity has been linked to a different proportion among several gut microbe phyla. Obese animals were the first to show evidence of changes to the main phyla, *Firmicutes* and *Bacteroidetes* [55,56,57]. In fact, initial studies using obese mouse models revealed a predominance of *Firmicutes* and a depletion of *Bacteroidetes* [55]. Other studies on leptin-deficient mice supported the increased *Firmicutes*/*Bacteroidetes* ratio in the gut microbiome [56,57]. However, another study has shown no changes to this variable and has even observed a lower *Firmicutes*/*Bacteroidetes* ratio in obese animals [29]. 

Several clinical investigations found that obesity-related intestinal dysbiosis in humans is characterized by a decrease in total bacterial diversity and richness and, for in vivo studies, by a shift toward a community composed of more *Firmicutes* and fewer *Bacteroidetes*. In a clinical study on obese Japanese patients, next-generation sequencing revealed that the ratio of *Firmicutes* to *Bacteroidetes* was substantially larger in the obese group compared to the group of non-obese controls, and some bacterial species were significantly associated with each group. In obese patients prevail: *Blautia hydrogenotorophica*, *Coprococcus catus*, *Eubacterium ventriosum, Ruminococcus bromii, Ruminococcus obeum*; in non-obese prevail: *Bacteroides faecichinchillae*, *Bacteroides thetaiotaomicron, Blautia wexlerae, Clostridium bolteae, Flavonifractor plautii* [58]. Indiani et al. found a similar pattern, which consists of an increase in *Firmicutes* and a decrease in *Bacteroidetes* in the gut microbiome of overweight children [59]. Further evidence suggests that the bacterial species *Eubacterium rectale*, *Clostridium coccoides*, *Lactobacillus reuteri, Akkermania muciniphila, Clostridium histolyticum*, and *Staphylococcus aureus* tend to increase in obese individuals [60]. The recent systematic review of Crovesy et al. revealed that obese individuals have higher levels of *Firmicutes*, *Fusobacteria*, *Proteobacteria*, *Mollicutes*, and *Lactobacillus* (*reuteri*), and lower levels of *Verrucomicrobia* (*Akkermansia muciniphila*), *Faecalibacterium* (*prausnitzii*), *Bacteroidetes*, *Methanobrevibacter smithii*, *Lactobacillus plantarum* and *paracasei* [61]. Another recent clinical investigation conducted in Italy, confirms an increase in *Firmicutes* and a decrease in *Bacteroidetes* in the gut microbiome of overweight/obese individuals, and that the relative abundance of certain bacterial taxa belonging to the *Enterobacteriaceae* family, which are known to possess endotoxic activity, was higher in the obese group compared to normal-weight controls [62]. Although the majority of clinical studies support the idea that a larger *Firmicutes*/*Bacteroidetes* ratio is a hallmark of obesity, some researchers have found no difference, and some suggest the contrary. However, these opposite results can be attributed in part to methodological variations. Schwiertz et al. discovered that the proportions of the genus *Bacteroides* were higher in overweight volunteers than in lean and obese volunteers and that the ratio of *Firmicutes* to *Bacteroidetes* shifted in favor of *Bacteroidetes* in overweight and obese participants [63]. Duncan et al. (2008) discovered that weight reduction did not affect the relative proportions of *Bacteroides* or the fraction of *Firmicutes* in the human stomach [64]. In another investigation, no significant changes in *Firmicutes*/*Bacteroidetes* ratios were seen between obese and normal-weight adults [65] or between obese and normal-weight children [66]. Moreover, a recent meta-analysis has demonstrated that the composition of *Firmicutes* and *Bacteroidetes*, as well as their ratio, do not distinguish lean from obese human microbiomes [67]. Anyway, many aspects of modern life, such as nutrition, exercise, food additives and toxins, antibiotic use, and overall level of physical exertion, all influence the microbiome found in the gut of obese individuals, in addition to methodological variations between the different studies, and that may explain a large amount of variation within populations in the abundance of the *Firmicutes* and *Bacteroidetes* phyla.

In summary, dysbiosis has been linked to NAFLD, but it is unclear whether this is a direct cause or simply a reflection of disease-associated abnormalities in the host’s immunological and metabolic systems. Moreover, the gut microbiome may vary throughout demographic groups and stages of NAFLD, although many of these variations can be attributed to the diverse molecular approaches used to classify bacteria to the species level and the varied methodology used to define NAFLD. Nevertheless, there is mounting evidence from both animal and human research that dysbiosis of the gut microbiome is crucial to the development and persistence of the condition.

## 3. Gut–Liver Axis and NAFLD

Several pieces of evidence have pointed out that gut microbiome dysbiosis has a prominent role in the malfunctioning of the gut–liver axis. The following paragraphs will examine the pathological mechanisms that dysregulate the equilibrium of the gut–liver axis and its relationship with the onset of NAFLD (Figure 1).

### 3.1. Intestinal Permeability: The Leaky Gut

The intestinal barrier allows communication between the gut and the liver [19,20]. Intestinal cells are connected by tight junctions (TJs), which are crucial for preserving the integrity of the intestinal barrier [68]. The leaky gut, characterized by impaired intestinal barrier function, is a well-documented hallmark of dysbiosis in patients affected by NAFLD [69,70]. Dysbiosis and subsequent impaired gut permeability lead to the increase of gut-derived toxins in systemic circulation constituting metabolic endotoxemia, which in turn contributes to the development of the chronic low-grade inflammation state observed in obesity and NAFLD [71]. Several in vivo studies have focused on the role of increased gut permeability in the pathogenesis of NAFLD and its relationship with liver inflammation and fibrosis. In leptin-obese mouse models, epithelial permeability was increased [72], and there was a shift in the distribution of the TJ proteins zonula occludens-1 (ZO-1) and occludin. Moreover, greater levels of proinflammatory endotoxins were found in the portal circulation [72]. The gene *F11r* (F11 receptor) in mice codes for junctional adhesion molecule A, a component of TJs; when active, it regulates intestinal permeability and inflammation by adjusting the function of the epithelial barrier [73,74,75,76]. Mice deficient in the *F11r* gene that were fed with a high-fat, high-fructose, high-cholesterol diet had severe steatohepatitis, as determined by the presence of histological signs of liver inflammation, fibrogenesis, and an increase in blood transaminases [77]. In vivo treatment of mice with dextran sulfate sodium (DSS) induced an increase in plasmalemma vesicle-associated protein-1 (PV1), a marker of endothelial permeability, and downregulated the intestinal TJ proteins ZO-1 and claudin [78]. DSS administration with a combination of a high-fat and high-fructose diet enhanced the disruption of the gut vascular barrier and aggravated hepatic inflammation and fibrosis in mice with NASH [79]. 

Bacterial overgrowth in the intestine and increased intestinal permeability due to leaky TJs have both been linked to the pathogenesis of NAFLD in humans [80]. A transmission electron microscopy study found that microvilli in the gut mucosa of adults with NAFLD were disorganized and TJs were widened [81]. Studying the intestinal absorption and urine excretion of orally-given chromium-51 labeled ethylenediamine tetra-acetic acid (^51^Cr-EDTA) provided strong evidence of enhanced intestinal permeability in patients with NAFLD [69]. The severity of liver steatosis was inversely correlated with the increase in ^51^Cr-EDTA excretion levels compared to healthy subjects. Moreover, patients with NAFLD had lower levels of ZO-1 expression in the duodenum compared to healthy controls [69]. A recent systematic review and meta-analysis have corroborated the relationship between increased intestinal permeability and the degree of hepatic steatosis in patients with NAFLD [82]. In children with NAFLD, changes to gut permeability have been linked to liver injury, and serum levels of lipopolysaccharide (LPS) were found to be considerably greater in children with NASH [70]. LPS can initiate a signaling cascade that ultimately leads to the phosphorylation and activation of a focal adhesion kinase (FAK) in the enterocytes [83] which, in turn, increases intestinal permeability [83] by modulating distribution of TJ proteins [84]. The protein zonulin has recently received increased interest because of its importance in maintaining the integrity of the intestinal mucosal barrier [85]. Zonulin may be released into the bloodstream in greater amounts in response to several conditions that compromise mucosal function [85]. The number of bacterial colonies in the large intestine was directly connected with the elevated serum levels of zonulin in obese people [86]. Pacifico et al. found that the severity of steatosis is linked to serum zonulin levels, which are much higher in obese people with NAFLD than in obese people with only obesity [87]. Studies in Turkey [88] and Italy [89] similarly showed that the levels of this protein were higher in children with NAFLD compared to the control group. Another study found that patients with NAFLD had significantly higher serum levels of zonulin and haptoglobin compared to healthy controls [90]. Zonulin is the progenitor of haptoglobin, a protein produced in the liver during the acute phase [91] and there is a functional connection between these proteins [85]. According to the results of a Japanese study including 126 patients with NAFLD, haptoglobin levels were significantly higher in those who met the histological criteria for a NASH diagnosis and were closely linked to hepatocyte ballooning [92].

According to the above findings from preclinical and clinical studies, there is reason to believe that repairing damage to the intestinal barrier could slow or stop the disease’s course.

### 3.2. Endotoxins and Inflammation

The population of anaerobic Gram-negative bacilli and the number of endotoxins they produce both increase in tandem with the deterioration of the gut microbiome. Overloading the liver with antigens such as endotoxins from the altered gut results in a loss of liver tolerance and the establishment of a proinflammatory milieu when the intestinal barrier is compromised. LPS is the main form of endotoxin produced by Gram-negative bacilli in the intestinal microbiome [93]. LPS is involved in the pathogenesis of liver inflammation [94,95]. Guo et al. found that continuous subcutaneous infusion of low-dose LPS in mice fed a regular diet induced hepatic steatosis and hepatic insulin resistance [96]. In the setting of high-fat diet-induced steatosis in mice, the administration of oral DSS caused an increase in portal LPS absorption, hepatic inflammation, and fibrogenesis [97]. 

Serum endotoxin levels, such as LPS, were shown to be higher in individuals with NAFLD compared to healthy controls [98,99]. It is demonstrated that in NASH patients the transition from simple fat deposition to steatohepatitis may involve endotoxins originating from the gut flora [99,100]. The presence of LPS in the systemic circulation is correlated with the severity of liver damage [101,102]. Patients with biopsy-proven NAFLD/NASH had higher levels of abnormal plasma immunoglobulin G against endotoxins, which were correlated with a greater degree of severity of NASH [103].

The inflammatory state induced by endotoxins is strongly linked to the immune response mediated by pattern recognition receptors (PRRs), primarily Toll-like receptors (TLRs), and nucleotide-binding and oligomerization domain (NOD)-like receptors (NLRs) in response to damage-associated molecular patterns (DAMPs) and pathogen-associated molecular patterns (PAMPs) [104,105,106]. TLRs are the primary microbe sensors on innate immune cells [105,106]. Due to their well-known role in the pathogenesis of NAFLD, TLR2, TLR9, and especially TLR4 have received significant interest [107]. Hepatocytes and Kupffer cells in the liver in particular express TLR4 in their plasma membranes [108]. Endotoxemia and increased expression of the TLR4 protein in the liver have been linked to the production of proinflammatory cytokines and systemic inflammation in patients with NAFLD and obesity [109,110]. Plasma endotoxin levels and hepatic TLR4 mRNA expression were found to be higher in NASH patients compared to NAFLD patients [111]. Moreover, an in vivo model showed that mice lacking TLR-4 have significantly less liver damage, inflammation, and lipid buildup compared to wild-type mice [112]. TLR4, when activated in response to endotoxins, triggers the production of signaling molecules like NF-kappa B. Liver injury occurs as a result of the release of inflammatory cytokines (TNF-α, IL-1, IL-6, IL-18), and fibrogenic cytokines/chemokines (TGF, MCP-1) [106,113,114,115]. NAFLD is closely linked to the increase, malfunction, and inflammation of adipose tissue, all of which are characteristics of obesity. Adipose tissue inflammation has been linked to LPS and other TLR ligands produced by the gut microbiome [116]. TLR signaling and inflammation in white adipose tissue were found to be higher in high-fat-fed mice compared to mice on an isocaloric fish oil diet [117]. Moreover, white adipose tissue inflammation was also prevented in genetically-altered mice lacking some TLR signaling system components [118]. 

Inflammasomes seem to be a key player in the activation of lipid peroxidation and the production of reactive oxygen species, two mechanisms that contribute to microbiome dysbiosis and the advancement of NAFLD [119]. The most extensively researched inflammasome in NAFLD is NLRP3 (nod-like receptor protein 3) [120]. Higher amounts of NLRP3 were identified in the livers of patients with NASH compared to those with simple steatosis, suggesting a role for inflammasomes in the progression of liver disease in NAFLD [121]. NLRP3 expression was found elevated in the liver of patients affected by NAFLD and it has been seen that its pharmacological inhibition prevents the progression of NASH [122].

Although there are still many issues to be answered before we have a complete understanding of how the gut bacteria, inflammation, and disease status interact, our expanding knowledge of this intricate interplay is creating new therapeutic opportunities for inflammation-dependent illnesses.

### 3.3. Metabolites Produced by the Gut Microbiome

**Figure 3:**
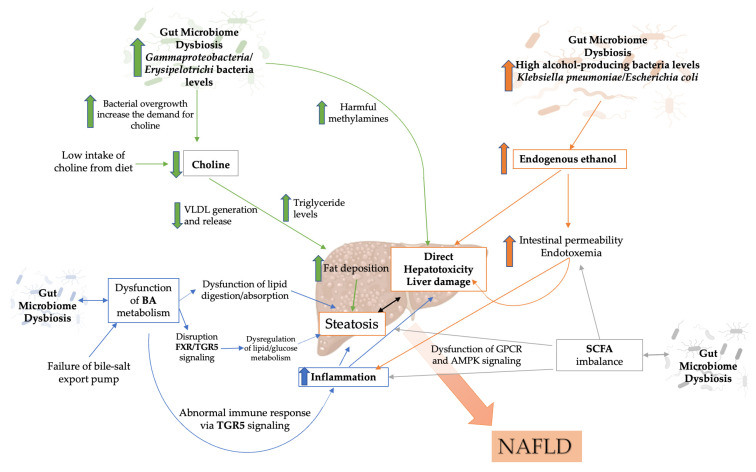
Schematic illustration of metabolites produced from the dysbiotic gut microbiome and involved in NAFLD pathogenesis. Created with https://BioRender.com (accessed on 29 October 2022) and modified with Microsoft PowerPoint v.16. BA: bile acid; SCFA: short-chain fatty acid; NAFLD: non-alcoholic fatty liver disease; FXR: Farnesoid Receptor X; TG5R: G-Protein-Coupled Bile Acid Receptor Gpbar1; GPCR: G-protein-coupled receptors; AMPK: AMP-activated protein kinase; VLDL: Very Low-Density Lipoprotein.

Metabolites are small chemicals formed as intermediate or end products of microbial metabolism and are one of the key ways in which the gut microbiota interacts with the host. These metabolites can originate either from the bacteria themselves, the microbial metabolism of food substrates, or the alteration of host molecules like bile acids. The dysbiotic gut microbiome produces microbial compounds that can negatively impact immune response and homeostasis, host energy metabolism, and mucosal integrity maintenance (Figure 3). In this section, we examine the role of gut microbiome-derived metabolites in NAFLD pathogenesis.

#### 3.3.1. Endogenous Ethanol

The hepatic damage of individuals with alcoholic liver disease and NAFLD is almost indistinguishable [123]. Blood ethanol levels have been reported to be elevated in human patients with NAFLD [124]. Among the gut microbiome, some microorganisms contain genes for ethanol fermentation from dietary carbohydrates [124,125]. The breakdown of ethanol into acetate and acetaldehyde causes the generation of fatty acids and liver oxidative stress, two major contributors to the development of NAFLD and NASH [126]. In addition to its direct hepatotoxicity, ethanol generated from the digestive tract has been shown to increase intestinal permeability and endotoxemia, both of which can exacerbate liver damage [127]. The amount of ethanol produced seems to be linked to the number of *Proteobacteria* (mainly *Klebsiella pneumoniae* and *Escherichia coli*) in the gut microbiome and the accessibility of carbohydrates from the diet [128]. Yuan et al. found that *Klebsiella pneumoniae* constitutes more than 60% of gut microbiome individuals with NAFLD in a Chinese cohort [116]. Moreover, they found that oral gavage or fecal microbiome transplants of *Klebsiella pneumoniae* into healthy mice induced NAFLD [128]. 

Therefore, inhibiting the growth of bacteria in the intestine that produces alcohol could be an encouraging strategy for treating NAFLD.

#### 3.3.2. Choline 

Choline, a phospholipid component of cell membranes, is essential for fat liver metabolism [129]. It contributes to the formation of very low-density lipoproteins (VLDL) and the transfer of lipids from the liver [130,131,132]. Choline deficiency can consequently result in fat deposition in the liver, as demonstrated in choline-deficient animal models of hepatic steatosis [133]. The key role of choline in the pathogenesis of hepatic steatosis in humans comes from indirect results, in fact, choline supplementation has been shown to reverse hepatic steatosis in patients receiving total parenteral nutrition [134]. In a clinical study conducted in China, the highest daily choline consumption of 412 mg was associated with a reduced risk of fatty liver disease in women with a BMI between 18.5 and 24.9, although, there was no observed link between choline consumption and a reduced incidence of fatty liver disease in obese or overweight women [135]. The production of VLDL may be influenced by choline [136,137]. Because choline is a precursor of phosphatidylcholine (PCH), and PCH is necessary for VLDL synthesis and excretion, triglycerides cannot be eliminated effectively from the hepatocytes in choline deficiency [138,139,140]. As a result, low choline levels lead to reduced VLDL generation/release, and elevated liver triglyceride levels [141]. A clinical study found a composition change in the intestinal microbiome in response to dietary choline intake with higher *Gammaproteobacteria* and *Erysipelotrichi* bacteria levels which correlated with liver fat alterations and choline deficiency [142]. Bacteria of the taxon *Erysipelotrichia* can convert choline to methylamines, which are toxic chemicals linked to liver damage [143]. In another clinical trial of 664 patients with NAFLD or NASH, postmenopausal women with inadequate choline consumption had more severe fibrosis [144]. A recent case–control study conducted in 2022 discovered that a high combined diet of choline and betaine was associated with an 81% decrease in visceral obesity-related fatty liver disease, although the optimal choline dosage remains unknown [145]. Around 10–15% of bacterial species require choline to produce phosphatidylcholine, a component of their membrane; hence, bacterial overgrowth may increase the demand for choline and lead to a choline shortage [146,147]. Thus, the gut microbiome can cause NAFLD by depleting choline and raising harmful methylamines [133,142,148], which can cause hepatic inflammation following absorption [149]. 

Gut microbes are known to convert choline to trimethylamine (TMA). TMA can be oxidized in the liver by hepatic monooxygenases to generate trimethylamine N-oxide (TMAO), which is then released into the systemic circulation [150]. Elevated levels of TMAO in the liver have detrimental effects on glucose homeostasis, increase insulin resistance, decrease glucose tolerance, and are implicated in atherosclerosis and other metabolic disorders such as obesity and NAFLD [151,152]. The link between biopsy-proven NAFLD and plasma concentrations of TMAO was recently supported by a study in which plasma concentrations of TMAO were associated with all-cause mortality in subjects with NAFLD, independently of traditional risk factors [153]. 

Therefore, the responses of the host and gut bacteria to dietary choline deficiency, as well as the production of bacterial biomarkers of fatty liver, corroborate the evidence that intestinal microbes are involved in NAFLD [133,142,148].

#### 3.3.3. Bile Acids

Cholesterol is the primary source of bile acids (BAs) in the liver. Primary BAs include chenodeoxycholic and cholic acids, and secondary BAs include deoxycholic and lithocholic acids [154,155]. After a meal, primary BAs are stored in the gallbladder, where they undergo further conjugation with glycine or taurine before being released into the intestine [154,155]. Due to their nature as amphipathic molecules with emulsifying activity, the primary role of BAs and their salts is to improve lipid and fat-soluble vitamin digestion and absorption [155]. The metabolism of BAs involves the gut microbiome [156,157]. The gut microbiome converts the primary BAs into the more hydrophobic secondary BAs via deconjugation and dehydroxylation [156]; these secondary BAs are reabsorbed in the distal ileum and transported back to the liver via the portal vein [157]. Deoxycholic acid and other BAs have antibacterial activity, preventing the overgrowth of bacteria in the small intestine and regulating the composition of the microbiome [158,159,160], while the microbiome can re-metabolize BAs into primary BAs and secondary ones [158,159,160,161]. 

Changes in the amount and composition of BAs have been linked to NAFLD. Alterations of plasma BA were observed across the whole NAFLD spectrum [162]. Serum levels of total BAs are commonly elevated in patients with NAFLD/NASH [163]. As liver fibrosis progressed, BA levels increased in a significant way [164]. In recent work, Wu et al. found that the serum BA profiles in type 2 diabetes mellitus (T2DM) patients with NAFLD were significantly different from T2DM patients [165]. Alterations in BA transporter expression in the liver and ileum contribute to NAFLD. When bile accumulates due to a decrease in flow its composition changes, which may contribute to liver damage [166]. The principal transporter of bile acids from the hepatocyte to the biliary system is the bile-salt export pump (BSEP) [167]. Cholestasis occurs when BSEP is faulty or mutated [167]. Studies of BSEP in NAFLD have found that decreased expression of bile-salt export pump (BSEP) is substantially connected with the severity of NAFLD and that overexpression of BSEP results in hepatic lipid accumulation [168].

Besides their involvement in lipid digestion and absorption, BAs also have a role in regulating hepatic lipid and glucose metabolism, through Farnesoid Receptor X (FXR) and G-Protein-Coupled Bile Acid Receptor Gpbar1 (TGR5) [169]. Increased insulin sensitivity and decreased hepatic gluconeogenesis and circulating triglyceride concentration are the results of BA binding to the FXR [170]. Dysbiosis in the gut microbiota may alter the ratio of primary to secondary BAs which, in turn, could disrupt FXR signaling resulting in significant effects on host metabolism [171]. An imbalance between secondary and primary BAs causes dysregulation of lipid and glucose metabolism [172] as a result of a reduction in signaling via the BA receptor FXR [173]. Reduced FXR activation also inhibits CYP7A1, a critical enzyme in regulating BA synthesis [174] and increases de novo lipogenesis and the synthesis of free fatty acids in the liver [175]. 

BAs can regulate glucose homeostasis and suppress the production of inflammatory cytokines by Kupffer cells [176,177] through the binding to TGR5 in the intestine and liver. TGR5 activation is also involved in the modulation of the microcirculation and fluid secretion in liver endothelium and biliary epithelial cells [178]. Moreover, in support of the important role of TGR5 receptor in the pathogenesis of NAFLD, it has been shown that in mice fed a high-fat diet the selective TGR5 agonist INT-777 lowers liver steatosis and related hepatocyte damage, as evaluated by plasma liver enzyme [179].

In summary, maintaining a healthy bile acid metabolism and gut microbiome can prevent the progression of NAFLD. In addition, the link between gut microbiota and BAs is an essential indicator for gut microbiota-targeted NAFLD therapy.

#### 3.3.4. Short-Chain Fatty Acids 

Some of the metabolites generated by gut bacteria have antagonistic effects. For example, unlike the ethanol produced by intestinal bacteria that may cause hepatic injury like alcoholic liver disease [180], short-chain fatty acids (SCFAs) are crucial for a wide variety of body processes, such as regulating appetite, metabolizing glucose and lipids, modulating immunity, and maintaining the integrity of the gut’s mechanical barrier [181]. SCFAs are also the primary source of energy for colonic epithelial cells, contributing to the maintenance of intestinal integrity. Therefore, supplementation with SCFAs has a beneficial impact on gastrointestinal disorders [182]. The gut microbiota produces SCFAs from indigestible starch and fiber in the diet; the specific SCFAs produced depend on both the microbiota in the gut and the type of fiber that is consumed [183]. Acetate, propionate, and butyrate constitute more than 90% of the SCFAs in the digestive tract [184]. After intestinal absorption, SCFAs are transported to the liver via portal circulation, where they serve as an energy source and play a significant role in lipogenesis and gluconeogenesis [185]. Beyond the beneficial effects, several studies [63,185] indicate that SCFAs are used as an energy source, hence enhancing adipogenesis in obese individuals. This may be affected by the individual SCFA, in fact propionate can inhibit de novo lipogenesis in the liver, whereas acetate can serve as a lipogenic substrate [35]. Furthermore, decreased levels of butyrate and butyrate-producing bacteria are often associated with metabolic disorders [186], indicating that the precise pattern of SCFAs may be essential to comprehending their role in obesity and NAFLD. Clinical studies have evaluated SCFA levels in the blood and fecal samples of patients with NAFLD. Bacteria producing SCFAs, such as *Bifidobacterium bifidum*, *Butyrivibrio*, *Megasphaera*, and *Prevotella*, are more prevalent in patients affected by NAFLD than in healthy controls, leading to higher concentrations of SCFAs in feces [187]. Moreover, patients with advanced fibrosis were shown to have increased fecal acetate levels, while patients with mild or moderate fibrosis had increased levels of butyrate and propionate [188,189]. In addition to functioning as metabolic substrates that charge hepatic glucose or lipid metabolism, SCFAs can also affect liver metabolism by acting as signaling molecules [190]. SCFAs may contribute in several ways to the onset of NAFLD. One possible pathogenetic mechanism involved the activation of G-protein-coupled receptors (GPCRs)-41 and -43 [191], which induce enteroendocrine cells in the mucosa to release the gut hormone peptide YY (PYY). PYY, in turn, slows intestinal transit time and increases nutrient absorption, resulting in lipid accumulation in the liver [191,192]. GPCR activation also stimulates GLP1, a peptide hormone that inhibits gastric emptying and food intake. GLP-1 also promotes hepatic lipid beta-oxidation and reduces hepatic steatosis [193]. Propionate and butyrate activate AMP-activated protein kinase to stimulate hepatic autophagy, a catabolic process that facilitates the hydrolysis of triglycerides and releases free fatty acids for mitochondrial β-oxidation [190,194]. 

There are several pieces of evidence that SCFAs are involved in the immune response and in fact, under certain circumstances, can stimulate the development of proinflammatory T cells like Th1 and Th17 [195]. Systemic acetate, interestingly, boosts the quick recall response of memory CD8+ T cells, leading to enhanced control of infection by intracellular parasites [195]. Moreover, several inflammatory cytokines, including TNF-α, a key mediator of liver inflammation and fibrosis, are also affected by SCFAs [196].

In conclusion, additional research is required to discover the major properties of SCFAs and their contribution to NAFLD pathogenesis in relation to gut microbiome dysbiosis, as well as to fully comprehend the molecular mechanisms and impacts of these compounds on the gut–liver axis.

## 4. Conclusions

For a long time, the existence of a large gut microbiome has been acknowledged, and awareness of the complexity of particular bacterial communities is growing. Furthermore, it is known that the microbiome is important beyond localized gastrointestinal disorders and can cause systemic alterations such as obesity and NAFLD. NAFLD has become a worldwide epidemic in large part due to lifestyle changes (excessive calorie consumption and decreased physical exercise). The prevalence of NAFLD is projected to quadruple by 2030 [197,198]. 

The gut and its microbiome, on the one hand, and the liver, on the other, interact in a feedback loop known as the gut–liver axis. It has been hypothesized that gut bacteria play a crucial role in keeping the gut–liver axis healthy. There is mounting proof that NAFLD is caused by a dysfunction in the gut–liver axis. Increased intestinal permeability and unrestricted transport of microbial metabolites into the liver are both effects of gut microbiome dysbiosis, which contributes to the development of NAFLD by dysregulating the gut–liver axis. Moreover, gut–liver axis dysfunction promotes NAFLD by altering hepatocyte lipid and glucose metabolism and disrupting the balance of inflammatory mediators.

Nowadays, the improvements in understanding the gut–liver axis are contributing to the creation of microbiota-based diagnostic, prognostic, and therapeutic tools for the treatment of NAFLD and other liver diseases. There is now considerable research aimed at determining which bacteria can be used as predictors of liver damage and NAFLD development. Recent studies are establishing the basis for microbiome-based therapy modalities, such as fecal microbiota transplant and probiotic therapies, which are gradually replacing observational studies in human patients [199,200]. To effectively translate and apply animal model findings to humans, however, well-designed, large-scale clinical trials covering numerous disease etiologies and patient ethnicities are required. Subtypes of NAFLD have been identified, and upcoming progress will depend on the systematic integration of their varying symptoms, gene expression discrepancies, gut microbiome, and metabolic differences. It will bring new insights and more precise treatment for NAFLD based on diverse phenotypes and the deployment of new technology to precisely intervene with specific gut bacteria. In the future, it will be important to understand whether changes in the gut microbiome and its metabolites in the context of the gut–liver axis are the causes or effects of NAFLD. Technological advancements and a better understanding of the gut–liver axis could stimulate research into gut microbiome-based medicinal approaches to improve the treatment of NAFLD.

## Data Availability

Not applicable.
